# Mechanical and Imaging Properties of a Clinical-Grade Kidney Phantom Based on Polydimethylsiloxane and Elastomer

**DOI:** 10.3390/polym14030535

**Published:** 2022-01-28

**Authors:** Izdihar Kamal, Hairil Rashmizal Abdul Razak, Muhammad Khalis Abdul Karim, Syamsiah Mashohor, Josephine Ying Chyi Liew, Yiin Jian Low, Nur Atiqah Zaaba, Mazlan Norkhairunnisa, Nur Athirah Syima Mohd Rafi

**Affiliations:** 1 Department of Medical Imaging, School of Health Sciences, KPJ Healthcare University College, Nilai 71800, Negeri Sembilan, Malaysia; izdihar.kamal@kpjuc.edu.my (I.K.); Ieqa_zz@yahoo.com.my (N.A.Z.); tirasyima@gmail.com (N.A.S.M.R.); 2 Department of Physics, Faculty of Science, University of Putra Malaysia, Seri Kembangan 43400, Selangor, Malaysia; josephine@upm.edu.my (J.Y.C.L.); yiinjian5447@gmail.com (Y.J.L.); 3 Department of Radiology, Faculty of Medicine and Health Sciences, University of Putra Malaysia, Seri Kembangan 43400, Selangor, Malaysia; rashmizal@upm.edu.my; 4 Department of Computer and Communication Systems, Faculty of Engineering, University of Putra Malaysia, Seri Kembangan 43400, Selangor, Malaysia; syamsiah@upm.edu.my; 5 Diagnostic Imaging Services, KPJ Seremban Specialist Hospital, Lot 6219&6220, Jalan Toman 1 Kemayan Square, Seremban 70200, Negeri Sembilan, Malaysia; 6 Institute of Advanced Technology, University of Putra Malaysia, Seri Kembangan 43400, Selangor, Malaysia; norkhairunnisa@upm.edu.my

**Keywords:** compression strength, effective atomic number, imaging properties, CT number, kidney phantom

## Abstract

Medical imaging phantoms are considered critical in mimicking the properties of human tissue for calibration, training, surgical planning, and simulation purposes. Hence, the stability and accuracy of the imaging phantom play a significant role in diagnostic imaging. This study aimed to evaluate the influence of hydrogen silicone (HS) and water (H_2_O) on the compression strength, radiation attenuation properties, and computed tomography (CT) number of the blended Polydimethylsiloxane (PDMS) samples, and to verify the best material to simulate kidney tissue. Four samples with different compositions were studied, including samples S1, S2, S3, and S4, which consisted of PDMS 100%, HS/PDMS 20:80, H_2_O/PDMS 20:80, and HS/H_2_O/PDMS 20:40:40, respectively. The stability of the samples was assessed using compression testing, and the attenuation properties of sample S2 were evaluated. The effective atomic number of S2 showed a similar pattern to the human kidney tissue at 1.50 × 10^−1^ to 1 MeV. With the use of a 120 kVp X-ray beam, the CT number quantified for S2, as well measured 40 HU, and had the highest contrast-to-noise ratio (CNR) value. Therefore, the S2 sample formulation exhibited the potential to mimic the human kidney, as it has a similar dynamic and is higher in terms of stability as a medical phantom.

## 1. Introduction

Medical imaging has proven its critical role in the healthcare field due to its ability to act as a valuable tool in diagnosis, therapy, surgical planning, postoperative assessment, and planning in radiotherapy treatment [1]. The increased volume of imaging procedures and the complexity of pathologies has led researchers to optimize the function at its highest standard performance. Often, the clinical diagnosis, which solely depends on a radiologist’s interpretation, takes time. A large volume of cases received daily tends to lead to errors, which may later cause misdiagnosis in patients [2]. This amenable side of medical imaging has been compensated with the introduction of quantitative imaging. The potential of quantitative imaging as biomarkers for many diseases such as brain ischemia, coronary artery disease, interstitial lung disease, and colorectal issues has become substantial in current radiology practice [3]. The growing need to employ quantitative imaging in clinical settings has motivated the study on the development of imaging phantoms [4,5]. For example, in the computed tomography (CT) examination, the standardization of CT pixel values with the Hounsfield Unit (HU) acts as vital measure to characterize tissue density, which is a vital component of quantitative imaging application [6,7]. This study presented the threshold values of radiation attenuation by the anthropomorphic phantom sample, including image quality, for a better understanding of the performance.

Briefly, the anthropomorphic phantom is critical in the quality control of modalities, as it allows the physicist to perform the calibration test, dose verification, teaching aid, and surgical guidance. The phantoms are usually made of materials that have human tissue equivalency and were designed with similar attenuation properties [3,8]. However, most of the designed phantoms face long-term stability issues. Usually, the agar and water-based gels phantoms demonstrate changes over time, due to continuous water expulsion and absorption cycles. Furthermore, the new invention of phantoms using the 3D-printing technique has limitations as well, including the lack of materials to mimic all the tissue properties and the high cost compared with other fabrication techniques [9].

The computed tomography (CT) scanner has been considered a gold-standard technique that has good accuracy, is faster in the detection of renal diseases, and has a much lower cost than the magnetic resonance imaging (MRI) scanner [10]. As the prevalence of renal diseases is often caused by incidental findings on abdominal CT images, an advanced approach is required to facilitate the early detection of renal diseases [11]. However, radiation exposure is still the primary concern as radiation is associated with radiation-induced toxicity, although this is underappreciated [12,13]. This study proposes the potential of a blend of Polydimethylsiloxane (PDMS) as a base of the kidney phantom material. PDMS has an elastic modulus of 360–870 kPa, which is larger than the elastic modulus of a real kidney [14]. Therefore, this research attempted to modify the PDMS with hydrophilic silicone and water, so that it can be a simple and economically affordable phantom with higher reproducibility of human tissue [15]. Additionally, this elastomeric polymer has ideal features such as nontoxicity, biocompatibility, blood compatibility, elasticity, transparency, and higher durability. There are several PDMS formulations, and those with Sylgard 184 are most often used in biological studies.

Clinically valid phantoms should demonstrate similar human tissue properties; hence, they should be designed with materials that have the same attenuation characteristics. For this purpose, the CT numbers and the image quality of the samples were evaluated to identify the relation of radiation properties and imaging properties to further validate the mechanical and chemical properties of the blended PDMS samples.

## 2. Materials and Methods

### 2.1. Fabrication of PDMS Phantom

The solid tissue-mimicking phantoms were fabricated from PDMS (Dow Corning, Michigan, US) with a standard density of 0.965 g/cm^3^ and viscosity (mixed) of 3500 cps. The base was a silicone-based polymer, belonging to the group of silicone elastomers. The typical mixing ratio for PDMS is a ten-part base and one-part curing agent (dimethyl vinylated and trimethylated silica, respectively) [16]. Different base/agent ratios of the PDMS network means a different amount of cross-linking [17]. Four samples were made with distinct material composition, as presented in Table 1, specifically: PDMS 100%, HS/PDMS 20:80, H_2_O/PDMS 20:80, HS/H_2_O/PDMS 20:40:40.

Initially, the elastomer base and the corresponding curing agent were weighed using an analytical balance, according to the predetermined weightage (Denver Instrument P-214, Brentwood, NY, US). Both solutions were then thoroughly mixed and stirred using a hot plate magnetic stirrer for five minutes. Before the mixture was poured onto a petri dish, a passivation layer (Ease Release 200) was applied to the petri dish to prevent strong adhesion between the mixture and the surface of the dish. The sample was then degassed in a vacuum desiccator for 30 min to remove any bubbles. The mixture was then heated in an oven at 80 °C for half an hour, and the temperature was increased to 100 °C for another one hour. Afterwards, the cured sample was left to cool at room temperature. After the PDMS network mold cured, the sample was stable and could be stored for months.

### 2.2. Compression Testing

**Figure 1:**
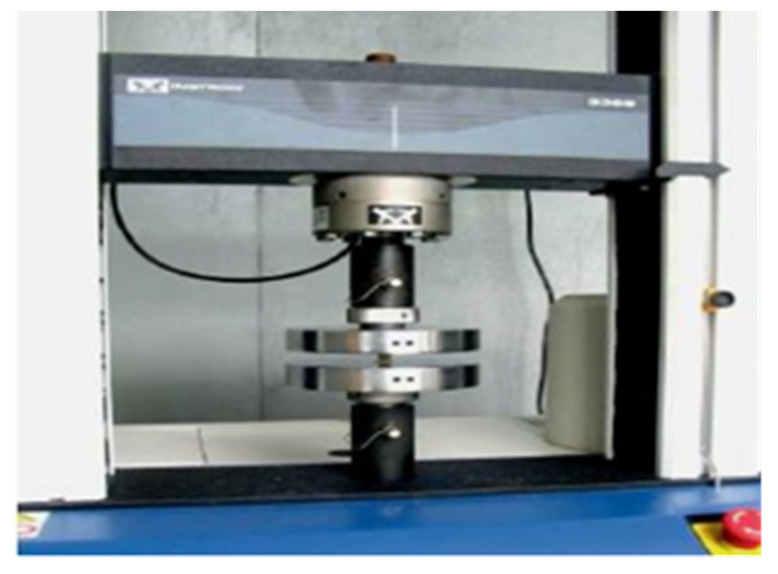
Instron universal compression-testing used for the compression test.

**Figure 2:**
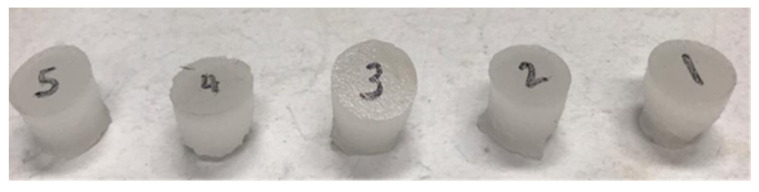
Cylindrical blend HS/PDMS 20:80 network for compression test.

The compressive test was performed by using an Instron universal compression-testing machine (Figure 1) with a 500 N load cell based on ASTM D1621 plastic compression testing. The phantom samples were first punched to cut the PDMS samples into cylindrical shapes. The initial dimensions were measured by a digital vernier caliper before testing, with an average value recorded from at least five measurements for each dimension: (i) 25.4 mm diameter and 25.4 mm height for cylinder-shaped compressive specimens. As shown in Figure 2, each sample was replicated 5 times in order to achieve the average reading for the compression test. The cylindrical samples were then placed on the compression testing plate, and the load cell was slowly lowered until it touched the surface of the sample. The contact between the sample and the plate was ensured by slowly moving the plate down until the machine detected the load. This test was conducted at a rate of 0.050 ± 0.010 in/min, as shown in Figure 2. From the stress–strain curve, the modulus of elasticity (MOE) can be calculated based on the following formula [14]:

(1) $$E=\frac{\sigma }{\varepsilon }$$

where *E* is the slope of the line in this region, and stress (*σ*) is proportional to strain (*ε*).

### 2.3. Radiation Attenuation Properties

In this study, we evaluated the tissue equivalence of the phantom by using a web-based software, Phy-X/PSD (Ataturk University, Erzurum, TR). The software is a web-based simulator that has a user friendly GUI interface, used to calculate Z_eff_ in the energy region of 1 keV–20 MeV for photons (both photon interactions and photon energy absorption) and in the energy region of 1 MeV–1 GeV for electrons, protons, alpha particles, and ions [18,19]. The tissue equivalence of the tissue substitutes is represented by this method for the computation of effective atomic numbers in this study. The methods for measuring Z_eff_ are discussed next. The first phase was concerned with the implementation of the chemical composition from the content to the software. The total weight number and fractions of the mole should be equal to 1 (or 100 percent). For example, sample 1 (mass) constituent fractions are 0.8 C_2_H_6_OSi + 0.2 C_7_H_22_O_2_Si_3_. Anything more may be measured at the same time as one item. The “+” sign can be used to incorporate other simultaneous material-Z_eff_ calculations. The software generated the interaction photon data in the energy regions for ions and charged particle interactions, pertinent to the context of medical physics (1 keV to 20 MeV for pho and 1 MeV to 1 GeV for tons of charged particles). The Z_eff_ in the case of continuous energy is defined as the output data.

### 2.4. Imaging Properties

In this study, the prepared phantoms were scanned using a 64-slice CT scanner (Somatom Definition Flash: Siemens Healthcare, Munich, DE) for the evaluation of imaging properties. Phantom samples were aligned and placed perpendicularly on the CT scanning couch before the scanning process. The scanning acquisition parameter was set accordingly with a detector configuration of 64 × 0.625, beam collimation of 0.5 mm, gantry rotation speed of 0.5 s, pitch of 1.375, and tube potentials of 80 kVp, 100 kVp, 120 kVp, and 140 kVp, with fixed-tube currents [20]. The images were reconstructed using a single filter kernel with 1 mm sections at 0.5 mm intervals.

**Figure 3:**
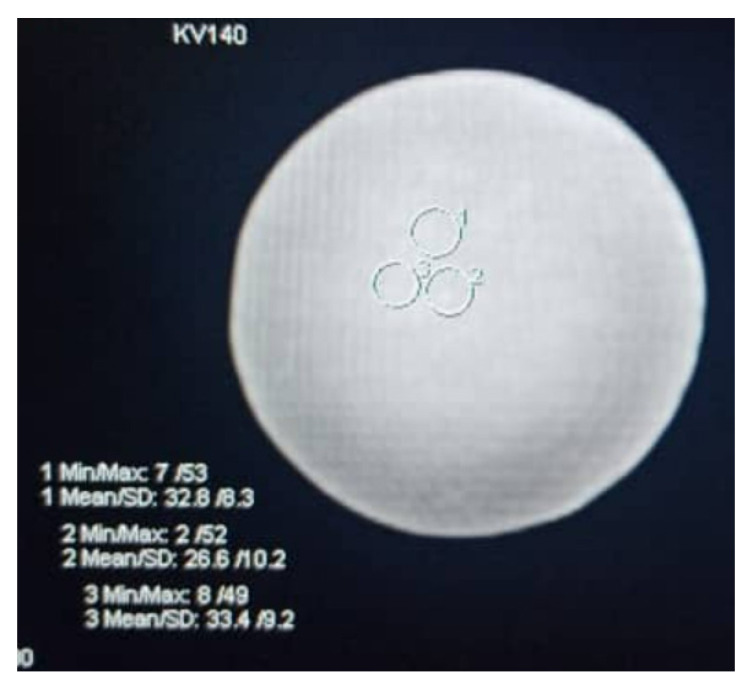
Example of objective image quality analysis to assess the CT number (mean) and image noise (SD) by putting the circular region of interest (ROI) in the middle of the sample.

After data images were obtained, the CT number of each sample was assessed and compared to that of human tissue features in CT scan images. The post-processing imaging tool was utilized to extract the CT numbers and noise values by placing the circular region of interest (ROI) in the selected image (Figure 3). The obtained value was then compared to the typical CT numbers of normal human tissue, particularly that of the kidney. The information regarding the CT numbers and noise was used to calculate the signal-to-noise ratio (SNR) and contrast-to-noise ratio (CNR) [21]. In a digital image, the signal is defined as a resultant exposure demonstrated on the image display monitor. Along with the exposure, there is an electronic noise that may be visualized along with the displayed image. The signal-to-noise ratio (SNR) is a method of measuring object detectability relative to noise in the digital image [21]. Higher image quality can be obtained by increasing the strength of the signal, which results in higher SNR than the amount of noise. Furthermore, the visibility of the anatomical details also depends on the SNR, where increasing the SNR increases the visibility of anatomic details and vice versa. Scientifically, the SNR in the region of interest (ROI) can be described as the ratio between the mean CT number of the ROI (*HU_ROI_*) and the associated SD of the ROI (*SD_ROI_*) expressed as [22]:

(2) $$SNR=\frac{HU_{ROI}}{SD_{ROI}}$$

and

(3) $$CNR=\frac{HU_{ROI}-HU_{B}}{SD_{B}}$$

where *HU_B_* and *SD_B_* are the background CT number and noise of the nearest region, respectively.

## 3. Results

Compression testing was performed on all the developed cylindrical phantom samples to characterize the compressive properties of materials. The samples yielded different compressive strengths and young modulus ranging from 0.41 to 4.06 MPa (Table 2). According to the table below, the HS/PDMS 20:80 sample had the highest compressive strength among all the samples.

Compressive strength was measured to determine the compressive force that the blended PDMS samples could withstand while retaining their shape. Changes were observed in all samples; however, the magnitude of this change varied. As can be seen from Table 2, the compressive stresses and young modulus were highest for sample HS/PDMS 20:80 (4.06 MPa) and the lowest value was recorded for sample HS/H_2_O/PDMS 20:40:40 as it crushed faster. This is due to less PDMS in the sample, whereas sample HS/PDMS 20:80 had the highest as the existence of HS and nil water. Silicone has a high atomic number; thus, it produced a stronger sample. Therefore, the mass fraction of silicone and water played a critical role in the mechanical properties of the sample [26]. On top of that, the strength of pure PDMS decreased by 30%, compared with the HS/PDMS 20:80 sample as HS was not added in the sample. Furthermore, the strength of the sample H_2_O/PDMS 20:80 was lower than pure PDMS by double, as water diluted the PDMS sample.

Based on the earlier analyses, HS/PDMS 20:80 is believed to be an optimum sample with the highest compressive strength. Therefore, further evaluation in terms of radiation attenuation properties was conducted for this PDMS sample, as this phantom will be used for dosimetry and calibration. The photon interaction with the matter was assessed based on the mass attenuation (μ/p) parameter. The mass attenuation coefficient (MAC), effective atomic number (Z_eff_), half-value layer (HVL), equivalent atomic number (Z_eq_), and linear atomic coefficient (LAC) of the HS/PDMS 20:80 sample are tabulated in Table 3.

Several works measured the LAC within the diagnostic radiology energy range, which was from 30 to 110 keV [27,28,29]. The calculated LAC for PDMS samples were compared with these previous works, and this comparison is shown in Table 4. This comparison shows that the values calculated in the present method agree with previous measurements and other theoretical data found in the literature for the energy range of 50 keV to 100 keV.

The HS/PDMS 20:80 sample was scanned using the Somatom Definition Flash with varying kVp values to correlate the relationship between different kVp and HU values. Table 5 tabulates the HU values at 80 kVp, 100 kVp, 120 kVp, and 140 kVp. The values varied from 14–130 HU, and specifically for 120 kVp, the CT number was 40–60 HU, within the range of the kidney’s CT number. The CT number decreased from 100 kVp to 140 kVp. As recommended by the American College of Radiology (ACR), a CT number study should be performed between 120 and 130 kVp [30]. Based on Table 5, at 120 and 140 kVp, the CT number was lower, and these values correspond to the values of the kidney, muscle, blood, and liver. Tailoring the concentrations of PDMS, HS and H_2_O would enable samples to be produced for specific parts of selected organs [31]. Additionally, Table 5 shows the contrast-to noise-ratio (CNR) and signal-to-noise ratio (SNR) for all tube potentials. Notably, the highest CNR occurring at 120 kVp was consistent and aligned with the ACR recommendation to choose a 120 kVp scan for a phantom study [6].

## 4. Discussion

According to Umale et al., the ultimate stress for human kidney varies from 2.8 MPa to 10.9 MPa, whereas this study reported 4.06 MPa for compression strength [8]. The elastic modulus of a real kidney was 180.32 ± 11.11 kPa (mean ± SD) and 95.64 ± 9.39 kPa under axial and transversal loadings, respectively. Information about the mechanical properties of human tissue will aid medical and biomechanical purposes to be used for diagnosis and simulation, respectively. The amount of water inside the sample may result in the fluctuation of the compressive strength over time, with the strength decreasing after weeks. The decrease in strength was prominent for higher amounts of water. Thus, it is advisable to fabricate a solid phantom that is not made of water-based material. On the other hand, the addition of HS increased the [SiR2-o-] backbone structure instead of the carbon backbone, thus adding photo-catalysts of the functional group, which can adhere and polymerize. As the HS can reduce the curing time, the polymerization process is controlled by the mass ratio between the amount of emulsifier, initiator dosage, HS oil, and the catalyst [32]. HS can form the PDMS to become gooey in texture with a soft structure. As the water and HS increase, the sample becomes more rigid and cloudy in appearance [31]. The addition of water induces moisture content into the phantom material. The moisture in the composites may interfere with the absorption effects by decreasing the effects of physical bonding and potentially acting as a lubricant between the polymer phases. A study by Sombatsompop and Sims (2004) suggested that to optimize the mechanical properties of polymers, the moisture content in the compound should be less than 1% [33].

X-rays and gamma rays are penetrating forms of high-energy electromagnetic radiation. The photon mass attenuation coefficient, effective atomic number, and half value layer are the basic quantities required for determining the penetration of X-rays and gamma rays in the matter [34]. The attenuation properties of this sample were studied to ensure that it could mimic the human tissue. Therefore, Phy-X/PSD software was utilized to calculate the Z_eff_ [35]. The effective atomic number was constant in the intermediate-energy region, whereas noticeable variation was observed for low (<20 keV), as well as high-energy regions. Overall, the PDMS Z_eff_ ranged around 4.00 to 5.00 in a higher energy range ≥ 1.00 × 10^−1^ MeV and was comparable with the previous work [19]. This Z_eff_ was lower at the intermediate energy range due to the dominance pair production and scattering effects. This sample showed a value of Z_eff_ close to the normal kidney value with a deviation of seven to fifteen percent, as compared to the pure PDMS from our previous study [11]. The interaction of X-/gamma rays with different materials can be measured by using HVL. HVL provides a clear indication of the thickness needed to attenuate the half strength of the gamma-ray incident, and reveal the X-ray penetration power. The HVL values increased with an increase in photon energy. Furthermore, it was also necessary to calculate the Z_eq_ factor for all materials to obtain the exposure buildup factor (EBF) and absorption process (EABF) [28]. It is vital to investigate the EBF and EABF as these characteristics will validate the material for medical application. The Z_eq_ values rose marginally at low energy levels and then increased significantly. The Z_eq_ value for this study was higher than the lung, adipose tissues, and muscle, yet lower than the bone as its Z_eq_ at 0.015 MeV measured 12.99 and rose till 14.12 at 1 MeV [36,37]. The chemical composition, Z_eq_, plays a crucial role in the buildup of gamma photons within the selected human organs/tissues.

CT was used to determine the X-ray attenuation coefficient, and the CT number of water was set at 0 HU [38]. For materials other than water and air, some dependence of the CT number on kVp was expected, primarily because of the photoelectric interaction’s strong dependence on both photon energy and atomic number. Consequently, the mass attenuation coefficient of each material is highly dependent on energy; as a result, the CT number depends on kVp as well. For lower energy scans, such as 80 or 90 kVp, photoelectric interactions would be expected to increase, particularly in high-Z material such as bone. Increased photoelectric interactions result in an increased measured CT number for high-Z tissues at low kVp, compared with the same tissues at higher energies. This supported the results for the sample containing HS and PDMS at the estimated ratio. Additionally, this aligned with Mustafa and Jackson, who showed that the CT number was dependent on the scan kVp, particularly for high-Z/high-density materials [39,40]. Furthermore, it was reported that bone CT number decreases with an increase in kVp.

As this CT number value fluctuates and is not constant, the image quality of the sample was evaluated. Based on Table 5, greater attenuation occurred at 120 kVp, reducing the signal received by the detector, which explained the lower SNR values. In a noisy image, the image contrast is low; this is caused by a large amount of background signal due to the Compton scattering effect, consequently reducing the CNR of the image [21]. This explains why the 140 kVp scan reduced the CNR by 90%, compared with the 120 kVp value. Therefore, any factor that affected the SNR affected the CNR as well.

As a result, the attenuation coefficient was highly dependent on the energy, and the photoelectric interaction was dominant at the lower energy range. In diagnostic imaging, the two predominant interactions between X-ray photons and matter were the photoelectric effect (complete X-ray absorption) and the Compton interactions (X-ray scatter with fractional loss of X-ray energy). There was a shift from a predominance of the photoelectric effect to the Compton interactions from the lower energy range of medical imaging (20–50 keV) to the higher energy range (50–150 keV), in which X-ray attenuation was strongly related to the electron density or mass of the image material, as used by the method proposed by Phy-X/PSD and verified by the CT material.

## 5. Conclusions

Based on the stability validation from our initial works, it was found that there was an urgency to fabricate a solid phantom that was not made of a water-based material. The effect of water and HS on the chemical properties of PDMS was not significant. In contrast to the radiation properties, the HS reduced the mass attenuation coefficient and X-ray attenuation coefficient of the pure PDMS, thus mimicking the CT number and effective atomic number of the human kidney. In this study, it is promising to suggest PDMS as the material of choice to be used as a stable kidney phantom in terms of good agreement with compressive strength and radiation attenuation. Furthermore, the blended PDMS imitates human tissue more precisely, and permits a wide range of possibilities for testing and phantom designs. Hence, it promises to be of value for use in both research and clinical settings, as a reliable and accurate tool for the development of phantoms, especially for in-vitro phantoms.

## Figures and Tables

**Table 1:** Chemical composition of the Polydimethylsiloxane (PDMS) sample.

Samples	Hydrogen Silicone (g) C_7_H_22_O_2_Si_3_	H_2_O (g)	PDMS (g) C_2_H_6_Osi
S1	0	0	20
S2	4	0	16
S3	0	4	16
S4	4	8	8

**Table 2:** Composition ration of each sample and compression strength.

Samples	Hydrogen Silicone C_7_H_22_O_2_Si_3_	Water H_2_O	PDMS C_2_H_6_Osi	Compression Testing (MPa)	Young Modulus (MPa)
PDMS 100%	0	0	20	2.6–2.8 *	2.61 *
HS/PDMS 20:80	4	0	16	4.06 ± 0.62	4.17
H_2_O/PDMS 20:80	0	4	16	1.09 ± 0.362	1.19
HS/H_2_O/PDMS 20:40:40	4	8	8	0.41 ± 0.05	0.516

* reported by other researchers [23,24,25].

**Table 3:** Radiation attenuation properties of HS/PDMS 20:80.

Energy (MeV)	Attenuation Properties				
	MAC (cm^2^/g)	Z_eff_	HVL	Z_eq_	LAC
1.50 × 10^−2^	4.586	11.38	0.15661	10.33	4.568
2.00 × 10^−2^	2.044	10.29	0.3514	10.43	2.037
3.00 × 10^−2^	0.736	7.91	0.97565	10.55	0.735
4.00 × 10^−2^	0.417	6.26	1.72455	10.62	0.416
5.00 × 10^−2^	0.300	5.34	2.3926	10.67	0.300
6.00 × 10^−2^	0.246	4.83	2.91631	10.7	0.247
8.00 × 10^−2^	0.198	4.35	3.62052	10.75	0.199
1.00 × 10^−1^	0.176	4.16	4.06981	10.78	0.177
1.50 × 10^−1^	0.150	4.00	4.79128	10.83	0.15
2.00 × 10^−1^	0.135	3.96	5.31868	10.85	0.136
3.00 × 10^−1^	0.116	3.93	6.18157	10.88	0.117
4.00 × 10^−1^	0.104	3.92	6.92316	10.89	0.104
5.00 × 10^−1^	0.095	3.92	7.59245	10.9	0.095
6.00 × 10^−1^	0.087	3.92	8.21515	10.91	0.088
8.00 × 10^−1^	0.077	3.91	9.35973	10.91	0.077
1.00	0.069	3.91	10.41402	10.91	0.069
1.50	0.056	3.92	12.79181	8.76	0.056
2.00	0.048	3.93	14.87477	8.48	0.048
3.00	0.039	3.99	18.42066	8.42	0.039
4.00	0.034	4.05	21.3392	8.39	0.034
5.00	0.030	4.13	23.78665	8.38	0.03
6.00	0.028	4.21	25.84345	8.38	0.028
8.00	0.025	4.37	29.07043	8.37	0.025
1.00	0.023	4.52	31.42182	8.37	0.023
1.50	0.021	4.87	35.01704	8.36	0.021

**Table 4:** LAC of HS/PDMS 20:80 and compared with other studies.

Energy (keV)	HS/PDMS 20:80	King et al. (2011) [27]	Aysun Böke (2014) [28]	Manjunath et al. (2015) [29]
30	0.710	0.389	0.383	0.384
40	0.402	0.279	0.277	0.278
50	0.290	0.246	0.233	0.241
60	0.238	0.218	0.213	0.215
80	0.191	0.201	0.191	0.196

**Table 5:** Average CT number, standard deviation (SD) and image quality of the samples.

kVp	Average CT Number	SD ROI	HU Background	SD Background	SNR	CNR
80	71	7.5	−802	10.3	15.12	87.11
100	63.7	6	−792	9.5	14.66	96.18
120	45	5.6	−789	8.2	2.56	100.9
140	30	8.3	−830	84.7	1.1	9.98

## Data Availability

Not applicable.
